# Emerging roles of mitochondria and autophagy in liver injury during sepsis

**DOI:** 10.15698/cst2017.11.110

**Published:** 2017-10-02

**Authors:** Toshihiko Aki, Kana Unuma, Koichi Uemura

**Affiliations:** 1 Department of Forensic Medicine, Graduate School of Medical and Dental Sciences, Tokyo Medical and Dental University, 1-5-45, Yushima, Bunkyo-ku, Tokyo, 113-8519, Japan.

**Keywords:** autophagy, mitophagy, non-canonical autophagy, sepsis, mitochondrial dysfunction, liver, immune response, secretion

## Abstract

Recent research indicates crucial roles of autophagy during sepsis. In animal models of sepsis induced by cecal ligation and puncture (CLP) or the systemic administration of lipopolysaccharides (LPS), autophagy is implicated in the activation and/or damage of various cells/organs, such as immune cells, heart, lung, kidney, and liver. Since sepsis is associated with an increased production of pro- as well as anti-inflammatory cytokines, it has long been considered that hypercytokinemia is a fetal immune response leading to multiple organ failure (MOF) and mortality of humans during sepsis. However, a recent paradigm illuminates the crucial roles of mitochondrial dysfunction as well as the perturbation of autophagy in the pathogenesis of sepsis. In the livers of animal models of sepsis, autophagy is involved in the elimination of damaged mitochondria to prevent the generation of mitochondrial ROS and the initiation of the mitochondrial apoptotic pathway. In addition, many reports now indicate that the role of autophagy is not restricted to the elimination of hazardous malfunctioning mitochondria within the cells; autophagy has been shown to be involved in the regulation of inflammasome activation and the release of cytokines as well as other inflammatory substances. In this review, we summarize recent literature describing the versatile role of autophagy and its possible implications in the pathogenesis of sepsis in the liver.

## INTRODUCTION

Autophagy, derived from the Greek ("auto" meaning "self" and "phagy" meaning "eat"), is a cellular system to degrade intracellular unnecessary materials, including proteins and organelles, for recycling 1. Until now, three types of autophagy have been reported; macroautophagy, microautophagy, and chaperone-mediated autophagy. However, molecular mechanisms and physiological implications of the latter two types of autophagy (microautophagy, and chaperone-mediated autophagy) are still obscure compared to macroautophagy. In this review, therefore, we feature only macroautophagy and hereafter refer to macroautophagy as autophagy. During autophagy, cytoplasmic materials are engulfed by newly synthesized vesicles, which are called isolation membranes or phagophores 2,3. Closed phagophores in which cytoplasmic constituents are included become to be double membrane structures called autophagosomes. Outer membrane of autophagosomes fuses with lysosomal membrane and resultant fusion structures are called autolysosomes. Cytoplasmic constituents within the inner membrane of autophagosomes are delivered into the luminal space of lysosomes, where hydrolytic enzymes such as cathepsins degrade them.

Autophagy is executed through a set of genes called atg (autophagy related) genes 4. Unc-51-like kinase-1 and -2 (ULK1 and ULK2), mammalian homologues of yeast Atg1, form a complex with Atg13, FAK family kinase-interacting protein of 200 kDa (FIP200), and Atg101 3. In healthy cells, this ULK complex resides in the cytosol and is inactivated by mammalian target of rapamycin complex (mTORC). Upon starvation, mTORC is inactivated and the ULK complex becomes activated to initiate the process of autophagy. One of the targets of ULK complex is class III phosphatidylinositol 3-kinase (PI3K) complex, which includes beclin-1 (Atg6) and Atg14. Class III PI3K complex facilitates the production of phosphatidylinositol 3-phosphate (PI3P) for autophagosome formation. Ubiqutin-like protein conjugation system is also involved in the process of autophagy 5. For example, after conjugation with the ubiquitin-like Atg12 protein, the Atg5 protein works as an E3 ubiquitin ligase to conjugate phosphatidylethanolamine (PE) to Atg8 5,6. Atg7 is an E1 ubiquitin-activating enzyme that activates both Atg8 and Atg12 5,6. These two ubiqutin-like conjugation systems characterize the process of autophagy.

By generating liver-specific atg5 or atg7 gene knock out (KO) animals, it has been demonstrated that basal autophagy is required to maintain liver homeostasis. Komatsu, *et al*. showed that in liver-specific conditional atg7 KO mice, starvation-induced autophagy is severely impaired 7. Conditional atg7 KO mice also develop a variety of degenerative syndromes, such as hepatomegaly in response to antibacterial drugs, steatosis, and the accumulation of protein aggregates resembling Mallory bodies 7. Development of multiple liver tumors has also been reported in mice with partial deletion of atg5 8. Anyway, autophagy protects hepatocytes and maintains homeostasis in both healthy and degenerating liver.

Sepsis caused by infection, trauma, or endotoxins leads to an excessive and uncontrolled immune cell response 9,10. Sepsis has an extremely high mortality rate with approximately 50% of patients delivered to the ICU (intensive care unit) with suspected sepsis eventually dying 11. During sepsis, the acute inflammatory phase leads to serious shock, which finally causes multiple organ failure (MOF) and subsequent mortality. Despite the self-explanatory role of inflammation in the pathogenesis of sepsis, there is also evidence against a critical role of immune reactions in the development of sepsis; whether or not there is a correlation between plasma levels of circulating cytokines and severity of outcome in patients suffering from sepsis remains a subject of debate 12,13. In addition, elevated blood cytokine levels usually resolve during the progression of sepsis.

Concurrent with the rising suspicion that uncontrolled immune responses might not play a central role in the pathogenesis of sepsis, mitochondrial dysfunction has come into the spotlight. Examinations of skeletal muscle biopsy samples from critically ill patients suffering from sepsis have shown that mitochondrial damage is more severe in patients who subsequently die than in those who eventually survive 14. Moreover, in contrast to the transient elevation in circulating cytokines, mitochondrial dysfunction persists even after cytokine levels return to basal levels 15. Given these indications, although cytokine production and mitochondrial dysfunction should be mutually connected, at least during the acute phase of the inflammatory response, the high mortality rate of sepsis might be attributable not only to the cytokine storm that eventually quiets down, but also to mitochondrial dysfunction that persists throughout the duration of the disease.

## MITOCHONDRIAL REPROGRAMMING IN MACROPHAGES DURING SEPSIS 

The molecular mechanisms underlying mitochondrial dysfunction caused by LPS (lipopolysaccharide)stimulation in the liver are poorly understood. On the other hand, a possible mechanism for mitochondrial reprogramming in immune cells has recently been proposed. Macrophages undergo metabolic reprogramming upon activation by immunostimulation such as LPS stimulation 16. Along with this change, "rested" macrophages become "activated" cells, in which cytokine production is activated. During this cellular transition from an anti-inflammatory to a pro-inflammatory phenotype, macrophages become more dependent on glycolysis rather than mitochondrial OXPHOS (oxidative phosphorylation) for survival 16,17. Hypoxia-inducible factor1α (HIF1α) is the inducible subunit of HIF1. HIF is a transcriptional activating complex that activates glycolytic genes, vascular endothelial growth factor (VEGF), and erythropoietin (EPO) in response to hypoxia. During this transition, the mitochondrial metabolite succinate accumulates in cells and stabilizes HIF1α by suppressing prolyl hydroxylase, which ordinarily leads HIF1α to proteasomal degradation in healthy cells 18. The stabilization of HIF1α in LPS-stimulated macrophages not only results in a cellular transition of metabolism, but also contributes to cytokine production by binding directly to the IL-1β promoter 18. Furthermore, increased succinate levels are thought to cause reverse electron transport at Complex I, which leads to ROS (reactive oxygen species) generation from the complex 19. ROS have been shown to be required for inflammasome activation and the subsequent production of inflammatory cytokines 20. Furthermore, increased succinate levels in LPS-stimulated macrophages contribute to inflammatory cytokine production by activating HIF1α as well as inflammasomes. Nevertheless, it remains to be examined whether this mechanism is also responsible for the generation of ROS in other cells. Furthermore, molecules that pave the pathway from LPS ligation to mitochondrial reprogramming are also poorly understood to date.

## AUTOPHAGY IN THE LIVER 

Direct evidence of autophagy in liver injury during sepsis has been reported by Watanabe, *et al.* 21. They surveyed liver samples from patients suffering from observable sepsis, as well as from CLP (cecal ligation and puncture)-induced sepsis model mice, and showed a massive accumulation of autophagic vacuoles in hepatocytes 21. Later, it was shown that autophagy plays a crucial role in protecting hepatocytes from septic insults. For example, in atg7 KO mice, liver injuries caused by LPS/D-galactosamine (GalN), a potent hepatotoxin 22, are more severe than in wild type (WT) mice 23. Likewise, it has been demonstrated that the systemic administration of LPS causes more severe damage in atg7-deficient liver than in WT liver 24.

Although its role in protecting hepatocytes against septic insult has been established, the cellular degradation activity of autophagy might not be fully activated in the liver during sepsis: several reports have pointed out perturbations in autophagy. There is a report indicating that in the hearts of sepsis model mice, autophagic flux is increased during the initial phase of sepsis, but declines at a later phase 25. The same research group has also reported similar results in the liver during sepsis 26. These observations coincide with the damage of lysosomes in LPS-stimulated cells. Nevertheless, other reports indicate the completion of the entire autophagy process in the liver of CLP-sepsis mice 27.

## INFLAMMATORY CYTOKINES AND SEPTIC LIVER INJURY 

**Figure 1:**
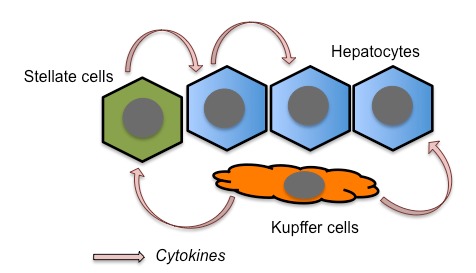
FIGURE 1: Release of inflammatory cytokines from hepatocytes, Kupffer cells, and stellate cells during liver inflammation caused by LPS. In the liver of mice systemically administered LPS, residential liver macrophages, Kupffer cells are activated to secrete inflammatory cytokines such as TNFα and IL-1β. These cytokines in turn activate stellate cells as well as hepatocytes. Both stellate cells and hepatocytes themselves are activated by LPS and subsequently release cytokines.

Several studies have proposed TNFα as a crucial mediator of liver toxicity during sepsis 23,24,28. Lower levels of TNFα secretion are observed in WT Kupffer cells, liver-residential macrophages, than in atg7-deficient Kupffer cells, suggesting that autophagy suppresses TNFα secretion from the cells 29. Therefore, this should represent one of the mechanisms by which autophagy ameliorates liver injury. Stellate cells are also responsible for the secretion of pro-inflammatory cytokines in response to LPS stimulation in the liver 30. Since hepatic stellate cells reside mainly along the sinusoids, these cells should participate in the earliest immune responses in the liver during sepsis. Hepatocytes can also secrete TNFα, at least in response to IL-1β stimulation 31. In addition, hepatocytes themselves express the LPS receptor, Toll-like receptor 4 (TLR4). Thus, TNFα secreted from Kupffer and stellate cells, as well as from hepatocytes themselves, should be responsible for hepatocyte injury (**Fig. 1**).

**Figure 2:**
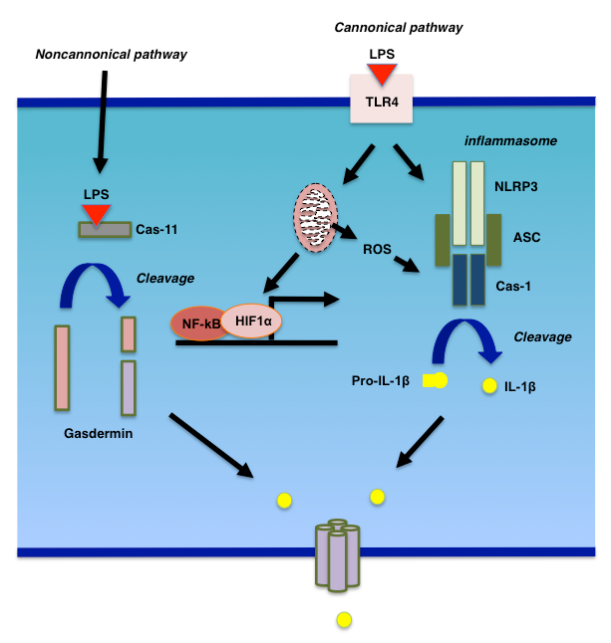
FIGURE 2: Cytokine secretion in response to LPS stimulation. There are two pathways for the LPS-initiated secretion of cytokines. In the conventional pathway, LPS binds to its cognate cell surface receptor, TLR4. The activation of TLR4 leads to the activation of NF-kB as well as HIF1α, the latter of which is mediated by mitochondrial dysfunction. Both NF-kB and HIF1α are involved in the expression cytokines as well as inflammasome components. Inflammasomes, comprising NLRP3, ASC, and Cas-1 for example, are involved in the activation of Cas-1. In an unconventional pathway, LPS binds directly to cytoplasmic Cas-11, which cleaves gasdermin to form a pore in the plasma membrane.

LPS binds to its cognate receptor, TLR4, and promotes the synthesis of cytokines, chemokines, and type-I interferons 32. TLR4 signaling is mediated by a number of adapter molecules, such as MyD88, TIRAP, TRIF, and TRAM. TLR4 signaling could be divided into MyD88-dependent and -independent pathways. For example, cytokine and interferon productions in response to LPS stimulation are mediated by MyD88-dependent and -independent pathways, respectively. There are at least four types of inflammasome, NLRP1, NLRP3, NLRC4, and AIM2 32. Activation of inflammasomes, such as the NLRP3 inflammasomes, for which the mechanism of activation is best characterized, occurs in at least two steps (**Fig. 2**). During the first step, NF-kB is activated to induce the expression of NLRP3 as well as cytokines. Then, in the second step, NLPR3 forms a complex, the so-called "inflammasome", with other molecules including caspase-1 (also called IL-1β-converting enzyme or ICE) and ASC. Indeed, it has been shown that LPS stimulation rapidly induces the expression of NLRP3 and other inflammasome components in hepatocytes 33,34. This induction is followed by the expression of TNFα as well as IL-1β, suggesting that hepatocytes produce inflammatory cytokines in response to LPS stimulation through the formation of inflammasomes 33,34. In parallel with inflammasome activation, LPS damages mitochondria as well as lysosomes. ROS from damaged mitochondria as well as cathepsins from lysosomes have been shown to be essential for inflammasome activation 35,36. Indeed, autophagy negatively affects inflammasome activation by eliminating ROS from mitochondria 37.

In addition to the canonical pathway for intracellular LPS signaling, which is initiated by the ligation of LPS to TLR4, LPS also binds directly to and activates cytosolic caspase-11 in mice (caspase-4 and -5 are the human homologues of murine caspase-11) 38,39. This pathway is a non-cannonical pathway that leads macrophages to a certain form of cell death called pyroptosis. During pyroptosis, caspase-1 cleaves gasdermin D (GSDMD) 40,41, and the cleaved GSDMD assembles on the plasma membrane to form a pore through which IL-1β or other inflammatory modulators, such as HMGB1, are secreted 42. Recently, it has been shown that gasdermin E (GSDME), also known as DFNA5, fulfills a similar role to GSDMD in non-immune cells 43,44. To obtain pore-forming status, GSDME is cleaved by caspase-3 rather than caspase-1 43,44. The role of gasdermins in LPS-stimulated liver injury is a subject awaiting examination.

## CARBAMOYLPHOSPHATE SYNTHASE-1 

Carbamoylphosphate synthase1 (CPS-1) is a urea cycle enzyme that catalyzes the generation of carbamoyl phosphate from ammonia and bicarbonate. CPS-1 expression is restricted to the liver and to some extent the intestine. In an experiment to find liver proteins whose expressions are dysregulated during sepsis, Struck *et al.*, using a comparative proteomics approach, found CPS-1 15. They also showed that plasma CPS-1 levels in baboons increase gradually over time following the injection of LPS. The kinetics of plasma CPS-1 levels differs sharply from that of TNFα: CPS-1 levels begin to increase several hours after LPS injection and continue increasing for the duration of the experiment 15. On the other hand, TNFα levels increase rapidly within several minutes of the injection and return to basal levels within several hours 15. Thus, when the transient increase in TNFα in circulation has resolved, CPS-1 levels in turn begin to increase. The increase in CPS-1 in circulation during sepsis should not derive from the enzymatic nature of this protein, but rather from its cellular localization: CPS-1 localizes to the mitochondrial matrix space. Indeed, Crouser *et al*. also confirmed the release of CPS-1 into circulation in CLP-sepsis model mice 45. They showed severe mitochondrial degeneration, such as decreased respiration and increased carbonylation of mitochondrial proteins 45. In their experiments, the increase of CPS-1 in the plasma took place much faster than that of alanine transaminase (ALT) during CLP-sepsis: peak CPS-1 levels were observed after 24 hours while ALT levels peaked after 48 hours of CLP injection 45. Thus, the occurrence of plasma CPS-1 should result from the release of liver mitochondria into circulation, not from hepatocyte necrosis. They also supposed the lysosomal clearance of damaged mitochondria through autophagy, so-called mitophagy 45.

CPS-1 release into the circulation prior to ALT during sepsis has also been confirmed in the LPS model 46. Furthermore, we have also shown that CPS-1 release from LPS-stimulated hepatocytes is associated with the release of mitochondrial, autophagosomal, and lysosomal proteins, confirming the lysosomal exocytosis of autophagosomes that include mitochondria 47. Lysosomal exocytosis also occurs in LPS-stimulated MEF cells, but not in LPS-stimulated atg5-KO MEF cells, further confirming the essential role of autophagy in this process 47.

## MODULATION OF LIVER INJURY DURING SEPSIS THROUGH AUTOPHAGY-MODULATING COMPOUNDS 

In accordance with the supposed perturbation of autophagy at least in the later stages of sepsis 25,26, a panel of autophagy-inducing compounds has been shown to ameliorate septic liver injury (**Table 1**). Carbamazepine (CBZ), which is an anti-epileptic drug that was recently identified as an autophagy enhancer, can alleviate liver injury in mice with CLP-sepsis 26. In CLP-mice, the bacterial loads to the liver and circulation are significantly suppressed by CBZ, suggesting the role of autophagy in bacterial clearance 26. Another report points to the importance of Ca^2+^-signaling in LPS-induced autophagy in the liver. Nicotinic acid adenine dinucleotide phosphate (NAADP) is a Ca^2+^-mobilizing molecule that is different from cyclic ADP-ribose (cADPR) and *myo*-inositol 1,4,5-triphosphate (IP_3_) 48. NAADP mobilizes Ca^2+^ from different intracellular storage sites, including ER and lysosomes 48, and intracellular levels of NAADP are regulated by CD38, an ADP-ribosyl cyclase. In the liver of LPS-induced sepsis model mice, it has been shown that CD38 KO results in decreased autophagy in response to LPS stimulation 49. LPS/GalN-induced liver injuries such as apoptosis and hemorrhage are also decreased in CD38-KO mice as compared to WT mice 49. An aglycone, genipin, which has been shown to reduce LPS/GalN-induced liver injury 50, was recently demonstrated to provide liver protection by up-regulating autophagy 51.

**Table 1:** Table 1. Chemicals that affect liver injury during sepsis by modulating autophagy. CoPP, cobalt protoporphyrin; SnPP, tin protoporphyrin

**Chemicals**	**Model**	**Cell/Tissue**	**Autophagy**	**Liver injury**	**Ref**
CoPP	LPS	Liver	↑	↓	53
Genipin	CLP	Liver	↑	↓	51
SnPP	LPS, CLP	Hepatocytes	↓	↑	54
NAADP	LPS/GalN	Hepatocytes	↑	↓	49
CBZ	CLP	Liver	↑	↓	25
Wortmannin	LPS/GalN	Liver	↓	↓	52

On the other hand, there are several reports demonstrating autophagy as an exacerbating factor in sepsis (**Table 2**). Wortmannin is a PI3K inhibitor that is frequently used as an inhibitor of autophagy 55 because PI3K is required in the early stages of autophagy 56. Liver injuries caused by LPS/GalN in mice have been shown to be alleviated by pretreatment with wortmannin 52: wortmannin alleviates not only LPS/GalN-induced increases in autophagy, but also cytokine production, ERK and JNK activation, and the death of hepatocytes. Deteriorated LPS-induced liver injury has also been reported in mice deficient in Kir6.2, an ATP-sensitive K^+^-channel 57. In this case, however, increased autophagy in response to LPS was observed in KO mice as compared to WT mice, suggesting that autophagy might be involved in liver injury rather than liver protection.

**Table 2:** Table 2. Release of intracellular contents from cells. nd, not determined

**Cell/Tissue**	**Model**	**Released content**	**Autophagy dependency**	**Cell death**	**Ref**
Hepatocytes	LPS	Mitochondria	Yes	No	47
Liver	LPS	CPS-1	nd	No	15
Liver	CLP	CPS-1	nd	No	45
Liver	LPS	Mitochondria	nd	nd	46
Jurkat, L929	TNFα	Mitochondria	nd	Necroptosis	61
Macrophages	LPS	IL-1β	Yes	No	58
Microglial cells	ATP	Autolysosomes	Yes	No	60
U2OS, etc	Anti-cancer agents	ATP	Yes	Apoptosis	59

## AUTOPHAGY-DEPENDENT AND -INDEPENDENT CASES OF INTRACELLULAR CONTENT SECRETION DURING IMMUNE RESPONSES

**Figure 3:**
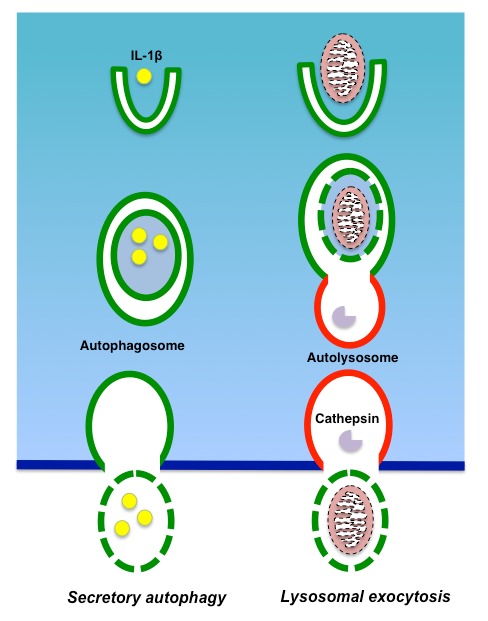
FIGURE 3: Secretion through autophagy machinery: secretory autophagy and lysosomal exocytosis. During secretory autophagy, autophagosomes do not fuse with lysosomes, but are delivered to the plasma membrane. During lysosomal exocytosis, autolysosomes fuse with the plasma membrane to extrude the contents.

Although autophagy is considered to be an intracellular degradation mechanism, recent research progress has shown it plays roles in other processes than the intracellular degradation of excess and/or unnecessary materials (**Fig. 3**). Unconventional functions of autophagy include a broad range of cellular activities, among which is the secretion of intracellular contents. Recent research suggests that autophagy plays important roles in septic liver failure, not only through intracellular degradation, but also via the secretion of cell contents.

TFEB, which has been identified as a transcriptional activator of lysosomal biogenesis 63, has also been found to be an inducer of autophagy 64 and lysosomal exocytosis 58. In healthy and/or nutrient-rich cells, TFEB is phosphorylated by the mTORC1 at the lysosomal membrane and retained in the cytoplasm 65. The cytoplasmic chaperone 14-3-3 is involved in this cytoplasmic retention by binding to phosphorylated TFEB 66. In unhealthy and/or starved cells, TFEB is activated through the release of calcium ions from lysosomes into the cytoplasm, a process that is mediated by the lysosomal membrane calcium ion channel, MCOLN1 64. A cytoplasmic calcium-dependent phosphatase, calcineurin, is responsible for the dephosphorylation of TFEB 67. It should be noted that MCOLN1 has also been suggested to be an NAADP-dependent calcium channel 49, although other channels have also been proposed as receptors for NAADP. It has also been shown that the CD38-catalized generation of NAADP and the resultant calcium mobilization lead to TFEB activation 49.

### Autophagy-dependent secretion of IL-1β

The secretion of IL-1β is one of the most important responses of immune cells during sepsis. Since IL-1β is a leaderless protein and is not secreted via a conventional secretary pathway, the mechanism of its secretion remains to be elucidated. IL-1β has been shown to be secreted by an autophagy-dependent mechanism under some circumstances 58. Further studies by the same group have identified TRIM16 as a receptor recognizing IL-1β-containing cargo as well as sec22b, syntaxin 3/4, and SNAP23/29 as components of the SNARE complex that mediates the docking of autophagosomes to the plasma membrane 68.

### Mast cell degranulation

Using bone-marrow-derived mast cells (BMMCs), Ushio *et al.* have shown that the ligation of the Fc receptor, which triggers the secretion of mediators of allergic responses such as histamine and leukotriene, is an autophagy-dependent process 69. They showed that although bone marrow cells derived from atg7-KO mice differentiate mast cells as WT cells do, mast cells derived from atg7-KO mice do not secrete granules in response to Fc receptor ligation, confirming autophagy-dependent degranulation 69. They also showed the co-localization of LC3-II and CD63, a marker of secretory lysosomes. Zhang *et al.* have also shown that mast cells stimulated by IgE secrete mitochondrial contents including mtDNA and ATP 70. They have shown that the addition of mitochondrial contents to mast cells in turn causes cell degranulation during which not only histamines and prostaglandins, but also IL-1β, TNFα, and IL-18 are secreted 70. Although they did not examine whether this process is dependent on autophagy or not, the secretion of mitochondrial components should occur during the degranulation of mast cells and be involved in allergic responses.

### Mitochondrial extrusion via an autophagy-independent pathway

Nakajima and colleagues showed that MEFs from c-Flip KO mice stimulated by TNFα extrude mitochondria from the cells 71. Prior to their release into the extracellular milieu, the mitochondria undergo fragmentation and cytoplasmic vacuolization is associated with the secretion 71. The cytoplasmic vacuoles seem to be derived from the plasma membrane since the membranes of the vacuoles were stained by the FM1-43 dye, which is incorporated into the plasma membrane. It has also been shown that z-VAD, cycloheximide, cytochalasin B, and pacilitaxel can all suppress the mitochondrial extrusion, suggesting that caspase activity, ongoing protein synthesis, actin and the tubulin cytoskeleton are all required for this phenomenon 71. Interestingly, 3MA, an inhibitor of autophagy, scarcely affects mitochondrial extrusion, suggesting that autophagy should not be involved in the process.

### ATP release from cells through autophagy-dependent lysosomal exocytosis

ATP is often released from damaged cells and acts in DAMPs (damage-associated molecular patterns) to activate immune cells. Tumor cells often show immunological responses, such as the release of ATP, during chemotherapy-induced cell death 72. Martins *et al.* showed that the secretion of ATP is executed through the exocytosis of lysosomes in which ATP is stored 59. Autophagy is involved in this ATP secretion since it has been shown that the inhibition of autophagy does not interfere with lysosomal exocytosis but does block ATP secretion 59.

### Autolysosome release from microglial cells

Takenouchi *et al.* have provided evidence for the release of autolysosomal contents from cells 60. In microglial cells stimulated by ATP through the purinergic receptor P2X7, they found that LC3-II levels increase over time 60. This increase cannot be attributed to the induction of autophagy because 3MA does not suppress the increase in LC3-II. Rather, they found that lysosomal activity is impaired. Lysosomal dysfunction might be attributed, at least in part, to the release of lysosomal contents from the cells. The content released from the cells consists not only of lysosomal luminal proteins such as cathepsins, but also autophagosomal proteins such as LC3-II. Thus, after fusion with lysosomes, autophagosomes are digested within lysosomes as well as secreted into the extracellular space.

### Release of mitochondria from TNFα-stimulated cells

Necroptosis is a form of programmed cell death that is executed by defined molecules such as receptor-interacting serine/threonine-protein (RIP) 1 and 3, and MLKL (mixed lineage kinase domain like pseudokinase), but morphologically resembles necrosis rather than apoptosis 73. Necroptosis occurs when cells do not undergo apoptosis for some reason, such as a lack of caspases. During TNFα-induced necroptosis, Maeda and Fadeel found that mitochondria are released from the cells into the extracellular space 61. Importantly, they showed that the released mitochondria are intact, as they contain mtDNA and stain positively with mitochondrial membrane potential-dependent dyes such as MitoTracker. The release of mitochondria is efficiently inhibited by the necroptosis inhibitor nec-1s, suggesting the involvement of necroptosis machinery in the secretion. They also showed that the exposure of monocyte-derived macrophages and dendritic cells to purified mitochondria results in the production of inflammatory cytokines including TNFα, IL-6, IL-8, and IL-10, confirming that the released mitochondria can act in DAMPs to activate immune cells.

## CONCLUDING REMARKS 

Many literatures have shown that autophagy is essential to maintain liver homeostasis under both healthy and disease conditions. Autophagy is consisted from multiple steps and the completion of the whole processes of autophagy is the prerequisite for its cytoprotective role. Although mitochondrial dysfunction and subsequent induction of autophagy to eliminate damaged mitochondria should be important to protect liver and other organs/tissues from septic injuries, the fate of damaged mitochondria might be dependent on lysosomes. There seems to be at least two destinations of mitochondria during sepsis: digestion within the cells and secretion into extracellular space. Factors determining the fate of mitochondria should be examined in the future studies. In addition, chaperone-mediated autophagy has also been shown to be necessary for liver homeostasis 74. Although the involvement of autophagy (macroautophagy) has been proved by use of conditional atg5- or atg7-KO mice in many studies, atg5/atg7-independent macroautophagy (alternative or non-canonical macroautophagy) has also been proposed 75. Thus, possible involvement of these types of autophagy in the septic injury in the liver should be examined in the future.

The extrusion of intracellular contents/substances from immunologically activated cells raises the question as to why cells actively secrete these contents. One explanation is that the intracellular capacity to degrade dysfunctional materials reaches its limit. Indeed, several studies have pointed out that the flux of autophagy is prone to stagnate in the liver during sepsis 26. An alternative explanation is that the cells extrude intracellular contents for specific purposes. For example, a number of studies have shown that autophagy-dependent secretion of intracellular materials, such as mitochondria, resulted in the activation of immune cells. Mitochondria, which have prokaryotic features such as CpG-methylated DNA as well as formylmethionine, act as DAMPs and have been shown to be involved in inflammation during sterile trauma 76. Indeed, several studies, including ours, have shown that purified mitochondria have the capacity to activate immune cells 47,61,76. Thus, the release of mitochondria from immunologically activated cells might be involved in the propagation of inflammation, which might be good or bad for human health depending on the situation. Further studies are needed to elucidate the exact roles of autophagy and mitochondrial release in liver injury during sepsis.

## Abbreviations

ALT: alanine transaminase
ATG: autophagy related
ATP: adenosine triphosphate
CLP: cecal ligation and puncture
CPS-1: carbamoylphosphat synthase
GSDM: gasdermin
IL: interleukin
KO: knock out
LPS: lipopolysaccharide
NAAP: nicotic acid adenine dinucleotide phosphate
PI3K: phosphatidylinositol-3-kinase
ROS: reactive oxygen species
TLR: toll-like receptor
TNF: tumor necrosis factor
ULK: Unc-51-like kinase
WT: wild type
